# Supplementary material for: Nonlinear ridge regression improves cell-type-specific differential expression analysis
Source: BMC Bioinformatics. 2021 Mar 22;22:141. doi: 10.1186/s12859-021-03982-3 (PMC7986289; doi:10.1186/s12859-021-03982-3)
Supplement: Supplementary file 4 — Additional file 4: Fig. S3. Precision (positive predictive value) for detecting cell-type-specific association in simulated data for gene expression of marker genes. [file 12859_2021_3982_MOESM4_ESM.pdf]

Figure S3

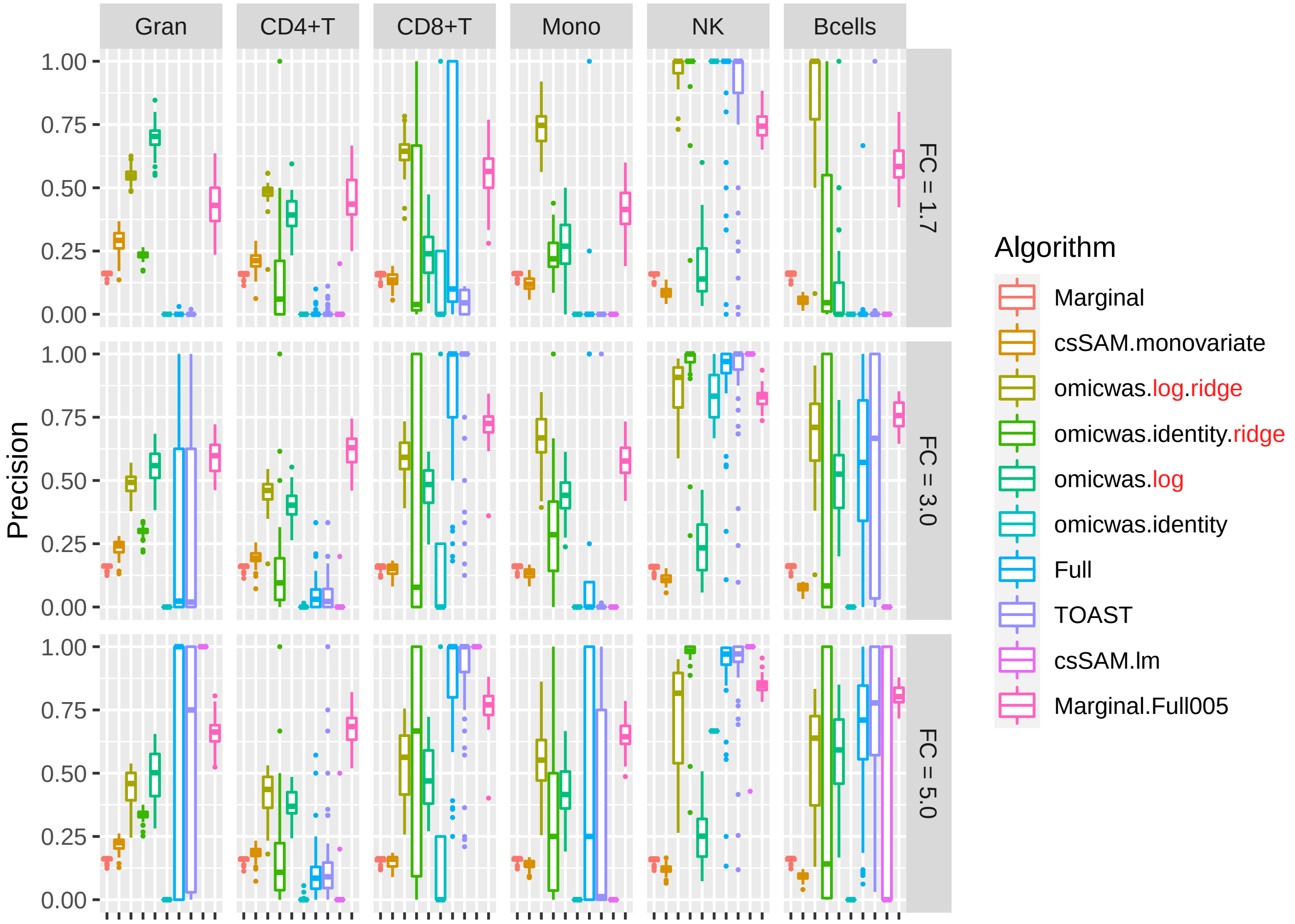

Precision (positive predictive value) for detecting cell-type-specific association in simulated data for gene expression of marker genes. The figure format is same as Fig. S1.
