# Supplementary material for: Nonlinear ridge regression improves cell-type-specific differential expression analysis
Source: BMC Bioinformatics. 2021 Mar 22;22:141. doi: 10.1186/s12859-021-03982-3 (PMC7986289; doi:10.1186/s12859-021-03982-3)
Supplement: Supplementary file 5 — Additional file 5: Supplementary note. Asymptotic distribution of ridge estimator. [file 12859_2021_3982_MOESM5_ESM.pdf]

Additional file 5: Supplementary note

Asymptotic distribution of ridge estimator

We here show that the ridge estimator $\hat{\boldsymbol{\theta}}(\lambda)$ is asymptotically normally distributed with the mean and variance in equations (11) and (12). Since the ridge estimator minimizes formula (10), its partial derivatives with respect to single parameters equal zero:

$$\left(\frac{\partial \boldsymbol{\mu}\left(\hat{\boldsymbol{\theta}}(\lambda)\right)}{\partial \boldsymbol{\theta}}\right)^{T}\left(f(Y)-\boldsymbol{\mu}\left(\hat{\boldsymbol{\theta}}(\lambda)\right)\right)-\lambda\begin{pmatrix} O & O & O \\ O & I & O \\ O & O & O \end{pmatrix}\hat{\boldsymbol{\theta}}(\lambda)=\mathbf{0}. \quad \text{(A1)}$$

The Taylor series of $\boldsymbol{\mu}$ and its Jacobian with regards to the variable $\hat{\boldsymbol{\theta}}(\lambda)$ centered at the true parameter value $\boldsymbol{\theta}$ become

$$\boldsymbol{\mu}\left(\hat{\boldsymbol{\theta}}(\lambda)\right) \approx \boldsymbol{\mu}(\boldsymbol{\theta})+\left(\frac{\partial \boldsymbol{\mu}(\boldsymbol{\theta})}{\partial \boldsymbol{\theta}}\right)\left(\hat{\boldsymbol{\theta}}(\lambda)-\boldsymbol{\theta}\right),$$

$$\left(\frac{\partial \boldsymbol{\mu}\left(\hat{\boldsymbol{\theta}}(\lambda)\right)}{\partial \boldsymbol{\theta}}\right) \approx \left(\frac{\partial \boldsymbol{\mu}(\boldsymbol{\theta})}{\partial \boldsymbol{\theta}}\right)+\left(\frac{\partial^{2} \boldsymbol{\mu}(\boldsymbol{\theta})}{\partial \boldsymbol{\theta} \partial \boldsymbol{\theta}^{T}}\right) \cdot \left(\hat{\boldsymbol{\theta}}(\lambda)-\boldsymbol{\theta}\right),$$

where the dot product of $(\partial^{2} \boldsymbol{\mu}(\boldsymbol{\theta}) / \partial \boldsymbol{\theta} \partial \boldsymbol{\theta}^{T})$ and the next term is taken by multiplying for each parameter and then summing up over parameters. We neglect the second or higher order terms of $\hat{\boldsymbol{\theta}}(\lambda)-\boldsymbol{\theta}$. By plugging into equation (A1), we obtain

$$\left(\frac{\partial \boldsymbol{\mu}(\boldsymbol{\theta})}{\partial \boldsymbol{\theta}}\right)^{T}\left(f(Y)-\boldsymbol{\mu}(\boldsymbol{\theta})\right)-\left\{\left(\frac{\partial \boldsymbol{\mu}(\boldsymbol{\theta})}{\partial \boldsymbol{\theta}}\right)^{T}\left(\frac{\partial \boldsymbol{\mu}(\boldsymbol{\theta})}{\partial \boldsymbol{\theta}}\right)-\left(f(Y)-\boldsymbol{\mu}(\boldsymbol{\theta})\right)^{T} \cdot \left(\frac{\partial^{2} \boldsymbol{\mu}(\boldsymbol{\theta})}{\partial \boldsymbol{\theta} \partial \boldsymbol{\theta}^{T}}\right)\right\}\left(\hat{\boldsymbol{\theta}}(\lambda)-\boldsymbol{\theta}\right)$$

$$-\lambda\begin{pmatrix} O & O & O \\ O & I & O \\ O & O & O \end{pmatrix}\hat{\boldsymbol{\theta}}(\lambda)=\mathbf{0}.$$

Thus,

$$\hat{\boldsymbol{\theta}}(\lambda)=Q(\lambda)^{-1}\, Q(0)\, \boldsymbol{\theta}+Q(\lambda)^{-1}\left(\frac{\partial \boldsymbol{\mu}(\boldsymbol{\theta})}{\partial \boldsymbol{\theta}}\right)^{T}\left(f(Y)-\boldsymbol{\mu}(\boldsymbol{\theta})\right)$$

$$=Q(\lambda)^{-1}\, Q(0)\, \boldsymbol{\theta}+Q(\lambda)^{-1}\left(\frac{\partial \boldsymbol{\mu}(\boldsymbol{\theta})}{\partial \boldsymbol{\theta}}\right)^{T} \boldsymbol{\varepsilon},$$

where $\boldsymbol{\varepsilon} \sim N(\mathbf{0}, \sigma^{2} I)$.
